# Low Resource Integrated Platform for Production and Analysis of Capped mRNA

**DOI:** 10.1021/acssynbio.2c00609

**Published:** 2022-12-10

**Authors:** Alison Obinna Nwokeoji, Tachung Chou, Eleojo Ahuva Nwokeoji

**Affiliations:** † Chemical and Biological Engineering, University of Sheffield, Sheffield S1 3JD, South Yorkshire, U.K.; ‡ School of Biosciences, University of Sheffield, Sheffield S10 2TN, South Yorkshire, U.K.; § All First Technologies, No. 208, Longnan Rd, Pingzhen District, Taoyuan City 324, Taiwan

**Keywords:** capped mRNA, mRNA biologics, integrated biomanufacturing platform, in vitro transcription, Vaccinia Capping System

## Abstract

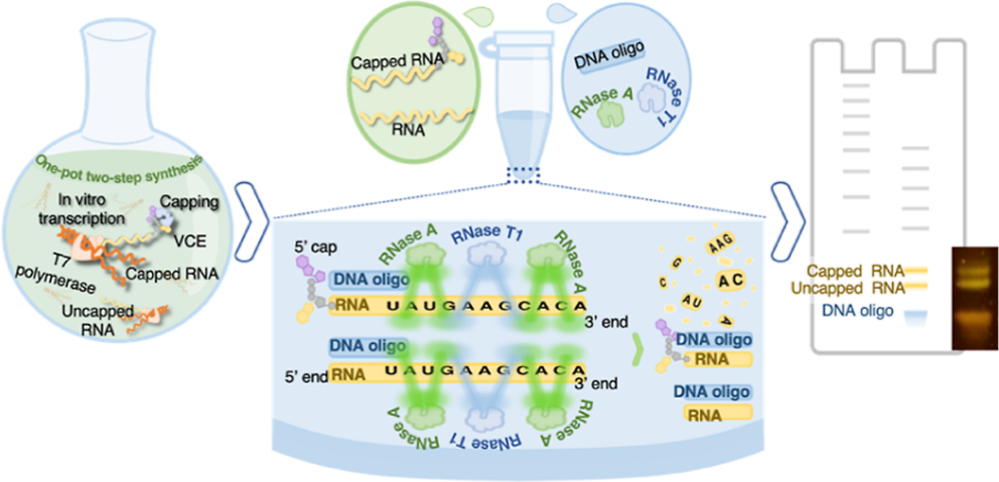

The existing platform for large-scale mRNA production is fast, but consumable costs, process technicality, and complexity represent key bottlenecks limiting global mRNA biologics manufacturing. Another challenge is the lack of a consolidated platform for mRNA product characterization and assays that meet regulatory requirements. Bridging these innovation gaps to simplify processes and reduce cost would improve mRNA biologics manufacturability, especially in low-resource settings. This study develops a “cotranscriptional” capping strategy that utilizes T7 RNA polymerase, and the *Vaccinia* Capping System to synthesize and cap mRNA. We created an “integrated reaction buffer” that supports both capping enzymes for catalytic and in vitro transcription processes, enabling one-pot, two-step capped mRNA synthesis. Additionally, we report a novel, one-step analytic platform for rapid, quantitative, capped mRNA analysis. The assay involves target mRNA segment protection with cheap DNA primers and RNase digest of non-hybridized or non-target sequences before analysis by single nucleotide-resolving urea-polyacrylamide gel electrophoresis (PAGE). The integrated approach simplifies production processes and saves costs. Moreover, this assay has potential applications for mRNA analyses and post-transcriptional modification detection in biological samples. Finally, we propose a strategy that may enable unparalleled sequence coverage in RNase mass mapping by adapting the developed assay and replacing urea-PAGE with mass spectrometry.

## Introduction

Messenger RNA biologics contain the blueprint of a viral, pathogen, or desired therapeutic protein; once introduced, the cell can produce this protein.^1^ The recent popularity of mRNA as a therapeutic candidate is due to its relatively short development times^2^ and relatively high safety.^3,4^ Capped mRNA is now the preferred and most potent active ingredient in most therapeutic candidates, as uncapped mRNA is less effectively translated and induces a detrimental innate immune response.^5−7^ In vitro transcription (IVT) is the primary platform for producing mRNA biologics and is capable of fast and large-scale manufacturing. However, the cost of raw materials, logistics, lack of infrastructure, and the complex production processes for mRNA biologics represent substantial challenges to increasing global manufacturing, especially in low-resource settings.^8−13^

One of the bottlenecks for large-scale mRNA biologic production is the cost associated with mRNA capping and the unavailability of reagents in large quantities.^14−16^ The capping residue, m7GpppG, was previously a popularly used cap analogue but lacks 100% capping efficiency and has the potential of generating 50% untranslatable mRNA due to the reverse orientation of cap.^17^ Alternatives such as the anti-reverse capping analogues (ARCA) are more effective in ensuring capped structures are in the correct orientation by preventing reverse incorporation of the analogue;^18^ however, ARCA significantly lowers transcript yield, and 100% capping is never achieved.^18,19^ Another option is the Trilink CleanCap, which was used in manufacturing the BioNTech COVID-19 vaccine.^20^ Despite advances, the required licenses for commercial synthetic 5′ cap analogues—expensive at scale—contribute to costs and hinder decentralized production of capped mRNA.

Another strategy deploys post-transcriptional capping, where the *Vaccinia* virus capping enzyme (VCE) adds cap (m7GpppN, also known as cap 0) to the purified IVT-generated mRNA, followed by its conversion to a cap 1 structure by *Vaccinia* 2′-*O*-methyltransferase.^18^ Moderna used this strategy in the pre-clinical development of their vaccine. A recent study has reported an efficient expression and purification protocol that enables large-scale production of *Vaccinia* enzymes, allowing scaled production of capped mRNA with 100% efficiency.^21^ The workflow in the *Vaccinia* strategy involves transcription, followed by DNA template decontamination and RNA purification before the capping process. Extra purification steps involved in standard IVT and capping strategy in capping increase production cost and time. A commercially available system, mScript, is reported to synthesize capped mRNA using a cocktail of T7 RNA polymerase, a trifunctional capping enzyme, a 2′-*O*-methyltransferase, and a poly(A) polymerase^18^ but also requires an RNA purification step. However, this system is expensive, and the additional therapeutic licensing required limits scaled production, especially in poorer countries.

Additionally, a strong imperative exists to develop simple, integrated, and cost-effective platforms for characterizing mRNA due to the lack of consolidated and transferable mRNA analytical tools that meet regulatory agency requirements.^22^ However, there are well-established methods for mRNA analysis in biological samples. One of those methods includes an RNase protection assay that requires a labeled RNA probe, purification steps, and autoradiography to detect mRNA.^23^ Detection and quantification of mRNA post-transcriptional modifications, including 5′ cap, poly(A) tail, and epitranscriptomes are also essential aspects of mRNA analytics. Traditional methods for 5′ cap detection rely on nuclease-mediated hydrolysis of mRNA to generate mononucleotides and subsequent detection of the incorporated P32 label at the 5′ terminal phosphate by polyacrylamide gel electrophoresis (PAGE) or high-performance liquid chromatography (HPLC).^24,25^ Another strategy is to cleave small sections of the 5′ end using RNase H combined with radiolabel detection by urea-PAGE.^26^ These approaches rely on autoradiography, which may not be ideal for industrial settings. Moreover, most of them are only suitable for detecting short RNA of about 50 nucleotide length.

Other strategies exist for label-free RNA analysis and capped detection. One method employs RNase H with a fused biotin-tagged RNA-DNA probe (to cleave the mRNA 5′ end), a purification step, and mass spectrometry to detect capping.^27^ With a cleavage site only 4–5 nt in length, the risk of generating additional fragments is high, thus impacting the method’s reliability. Other recent methods include a reportedly improved Rnase H method,^28^ ribozymes cleavage-based assays,^29^ or biosensors to detect mRNA cap structures.^30^ Although these approaches yield valuable, semi-quantitative or quantitative information and avoid radiolabeling, reagent or process costs may limit their industry applications, especially in low-resource settings.

With the increasing need for equitable, global, and decentralized access to biologics, there is a growing demand for simple, rapid, flexible, and scalable biomanufacturing and bioanalytic systems that will enable affordable, safe, and consistent production of biologics.^31^

To address this demand for more straightforward and affordable biomanufacturing platforms for mRNA therapeutics, we developed an integrated, single-pot platform that allows for simultaneous IVT and capping of mRNA mediated by T7 polymerase and *Vaccinia* capping enzymes, respectively. The system’s essential components are T7 polymerase, *Vaccinia* capping enzyme (D1 and D2 subunits), nucleotide triphosphates (NTPs), *S*-adenosyl methionine (SAM), and an optimized buffer compatible with both enzymes. Enzymatic post-transcriptional capping eliminates the need for patented synthetic cap analogues, the ARCA system, or expensive commercial capping alternatives. In addition, it circumvents the need for multiple purifications following IVT and before mRNA capping used in conventional mRNA platforms. We demonstrate that this system produces yield comparable to the commercial IVT kit and has 100% capping efficiency.

In addition, we develop a simple and rapid urea-PAGE method for detecting and identifying synthesized mRNA products and quantitative detection of capping. This method utilizes inexpensive DNA primers or probes of 16–25 nt length to protect the 5′ end of mRNA before digestion by single-stranded RNA-specific RNases (RNase A and RNase T1), followed by analysis by denaturing urea-PAGE. A novel feature of our method that distinguishes it from other methods is that it utilizes inexpensive DNA probes and a cheap sensitive dye. In contrast to the original RNase protection assay, which uses labeled RNA probes, our method employs unlabeled, inexpensive DNA probes to create DNA–RNA hybrids resistant to RNA cleavage. Moreover, this method requires no biotin tagging, autoradiography, or prior purification of IVT material before analysis. We also propose that the developed assay could adapt HPLC–mass spectrometry analytical approaches to identify mRNA and post-transcriptional modifications.

## Results and Discussion

### Simple Empirical Optimisation of a Single Reaction Buffer for In Vitro Transcription and Capping Reactions Mediated by *Vaccinia* Enzymes

To integrate the IVT and *Vaccinia* enzyme-mediated capping into one process, we investigated their reaction buffer compositions established in the literature and assessed each component’s concentration versus activity profile, essentiality, and compatibilities. Analysis of the data in the literature reveals that *Vaccinia* capping and IVT buffers have a combined total of 11 chemicals but only share four components in common, Tris-HCl, MgCl_2_, DTT, and KCl (Table S1). The KCl appears once out of the 11 transcription buffers in the literature/manufacturers’ manual. Some chemicals were essential for capping but not for IVT and vice versa. Therefore, to design an integrated buffer system compatible with IVT and capping reactions, it was necessary to consider the potential impacts of the chemical components on each reaction buffer. For the integrated buffer composition, we selected the final chemicals and concentrations by subjecting them to the following formulated rules: (1) include the chemical if it was essential for one reaction type even if it is not present in the other. It is essential when a chemical occurs at 100% frequency in the buffer composition found in the literature. (2) exclude if a chemical is not essential and not found in the other reaction. (3) Rule of integration: Use the highest concentration value for chemicals that occur in both unless it is detrimental to the other buffer or if evidence exists that the lowest quantity has the same effect as the highest value, in which case use the lowest value.

On this basis, we eliminated NaCl, Triton X-100, and Tween-20. The removal of these chemicals was precautionary as their effects on *Vaccinia* capping enzymes is unknown in the literature and is possible that it is not particularly essential to T7 polymerase activity. A study has shown that elevated NaCl concentrations are detrimental to T7 polymerase,^32^ but inhibitory concentration for *Vaccinia* enzymes is unknown. Moreover, the storage buffer of T7 RNA polymerase and *Vaccinia* capping enzymes have a high concentration of NaCl (100 mM), so IVT will still benefit from residual salt from these.

**Figure 1:**
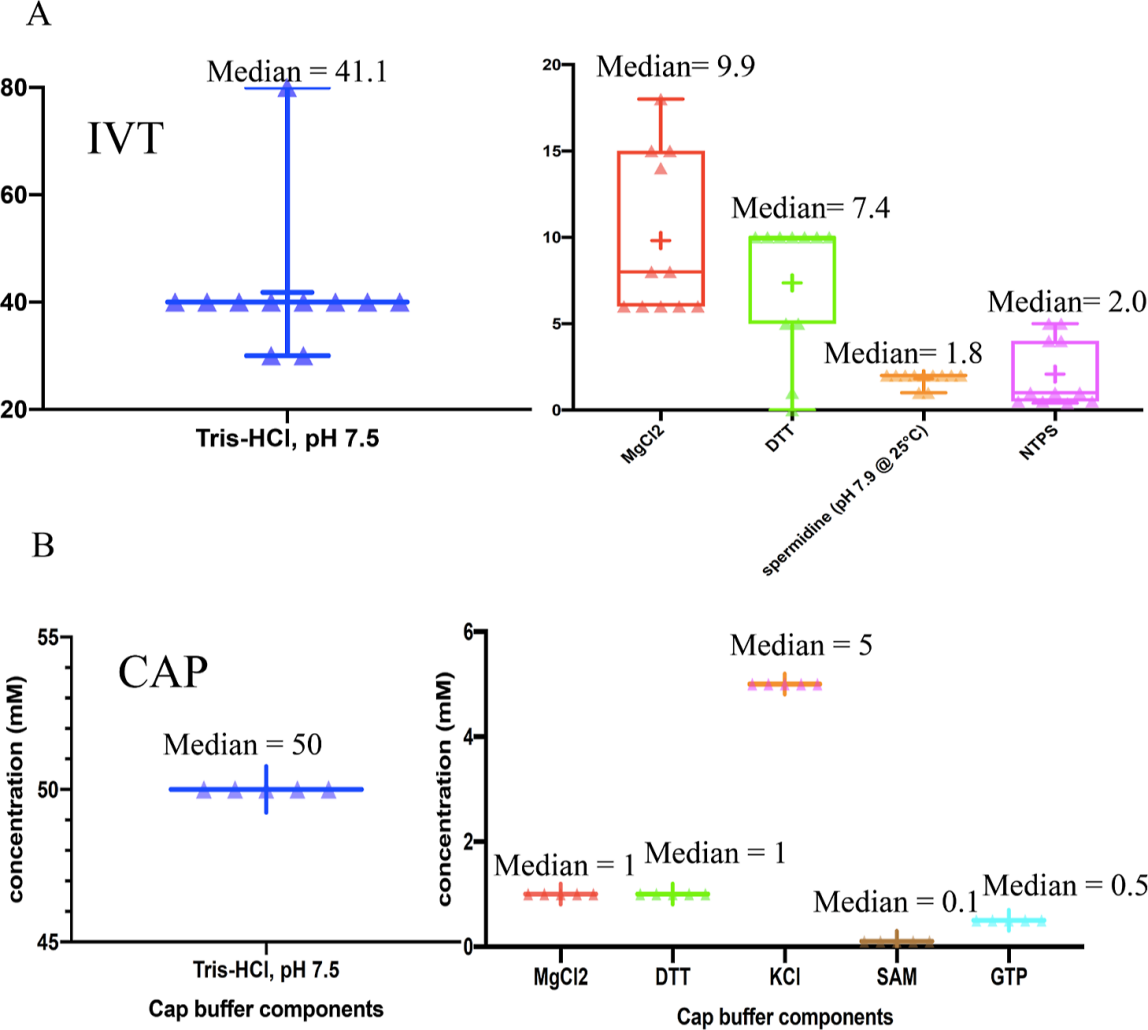
Analysis of the similarities and differences between capping and IVT buffer. Mean concentration of IVT and cap reaction buffer components used in the literature. (A) Box and Violin plot showing the chemical composition (and their concentrations) of IVT reaction buffers from the literature references. Median concentrations are indicated in the chart. (B) Box and Violin plot showing the chemical composition (and their concentrations) of capping reaction buffers found in the literature.

Further analysis of literature data to predict optimal chemical concentrations reveal a median of 41.1 mM for Tris-HCL, 9.9 mM MgCl_2_, 7.4 mM DTT, 1.8 spermidine, and 2.0 mM NTPs for T7 polymerase (Figure 1b; Table S1). For *Vaccinia* capping enzymes, all buffers found in the literature contain identical concentrations of chemicals (Table S1). We then applied rule 3 to generate chemical concentrations for the predicted integrated buffer (see Table S2).

### Efficacy of the Integrated Buffer System for Long and Short mRNA Synthesis

**Figure 2:**
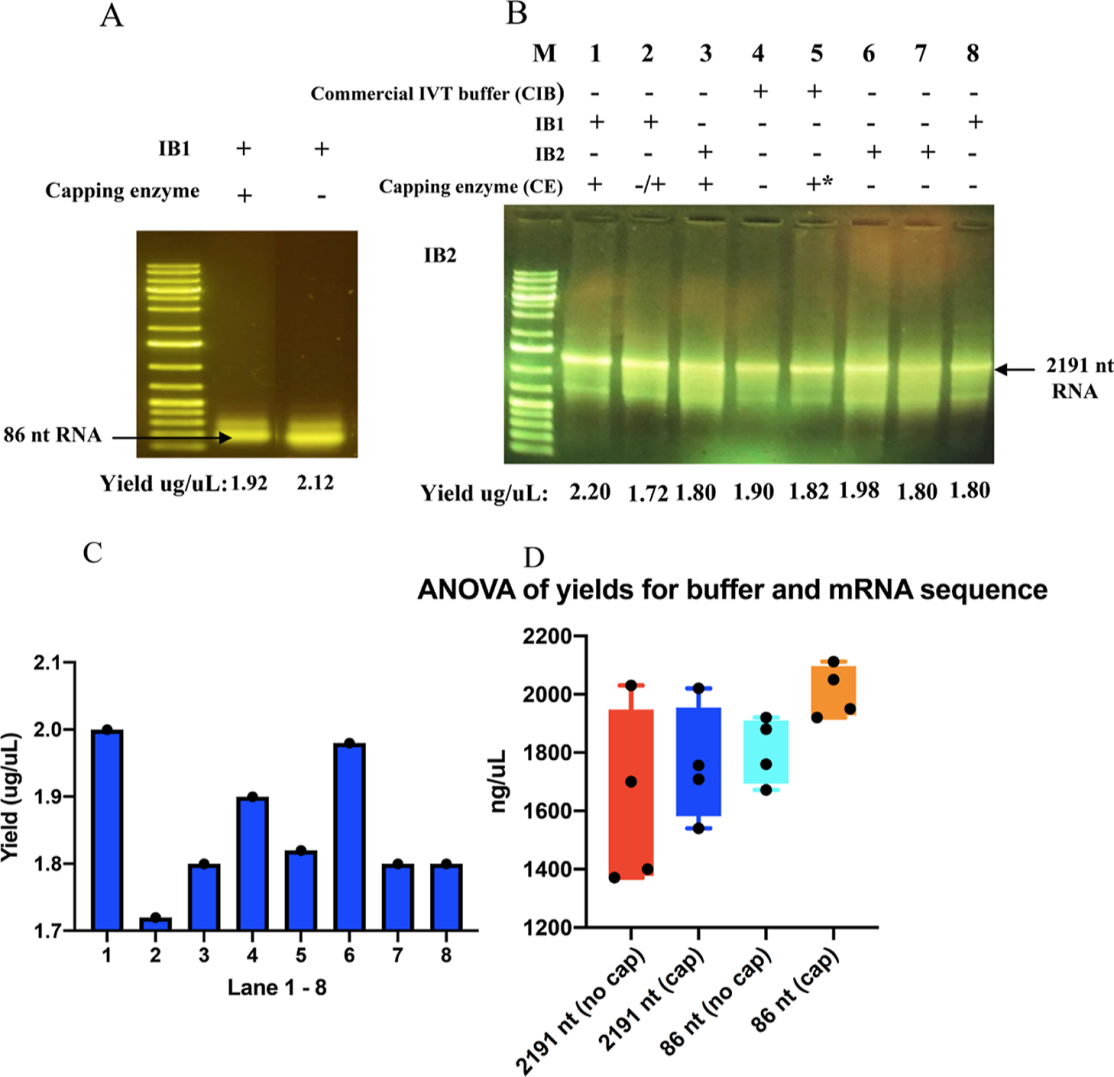
Agarose gel analysis comparing RNA generated from different formulated IVT reaction buffers and conditions. + and – means reaction components are present or absent, respectively. ± means that the component was absent initially but added later. +* means that capping substrates (SAM and GTP) were added later in the reaction. (A) DNA template for 86 nt RNA was transcribed in IB1 buffer, with or without capping enzyme added, and the product was analyzed by 1% agarose gel electrophoresis. (B) DNA template for 2191 nt RNA was transcribed commercial and integrated IVT buffers under the conditions indicated by gel lane labels, and the product was analyzed by 1% agarose gel electrophoresis. The yield of each sample was determined by spectrophotometric analysis (nanodrop) given at the bottom of each lane. (C) Bar chart for yield obtained for CIB, IB1, and IB2 shown in agarose gel in panel B. (D) Statistical graph comparing titers of purified 100 μL IVT mRNA solution (obtained from an integrated buffer containing (cap) or lacking (no cap) the *Vaccinia* capping enzyme.

Following the rational design of an integrated buffer, it was necessary to experimentally test the performance of the integrated buffer and how it affects mRNA yield. First, we wanted to know the effect of the capping substrates [guanosine 5′-triphosphate (GTP), SAM] and enzymes on the IVT. Therefore, we set up an IVT reaction in the integrated buffer with or without capping enzymes. We transcribed a DNA template encoding an 86 nt RNA in these buffers. Following the IVT reaction, an optimized method using DNase I treatment in conjunction with solid phase extraction using a silica membrane spin column to purify further the RNA product was performed before agarose gel electrophoresis and nanodrop spectrophotometer analysis. The agarose gel analysis shows (Figure 2A, lanes 1 and 2) that an 86 nt RNA was synthesized in the presence of all components (integrated NTPs, T7 polymerase, DNA template, and capping components) with or without the capping enzyme. The spectrophotometric analysis shows that the reaction buffer with capping enzymes had only a slightly higher RNA titer (2.12 μg/μL) than the one with the enzyme component (1.92 μg/μL) (Figure 2A, lane 2). This result shows that capping substrates and the *Vaccinia* Capping System do not inhibit but support IVT.

We then tested the synthesis of a longer mRNA (2191 nt) in formulated integrated buffers (IB1 and IB2) and other varied conditions, including introducing a capping enzyme at different reaction stages. The generated products appear heterogeneous as two RNA bands appear on the agarose gel. The shorter RNA may be due to incomplete transcription driven by sequence composition. Although it is hard to say without sequencing, the polyA tail or some secondary structure present in this 2191 nt RNA may be impacting the processivity of the T7 polymerase resulting in a fraction of the transcripts being shorter. Initial analysis of the RNA concentration shows that reaction performed in integrated buffer 1 (IB1) containing capping enzymes appears to have a similar or higher RNA titer (2020 ng/μL) (Figure 2B, lane 1) than other conditions, including reactions with commercial buffer (CB) and integrated buffer 2 (IB2) with or without capping enzyme (1800–1980 ng/μL) (Figure 2B, lanes 2–8 and Figure 2C). IB2 stock buffer is mainly identical to IB1 except for containing 20 mM GTP and the absence of SAM and VCE (during the reaction). IB2 is used as a control for comparison to assess the potential impact of core capping components (SAM, VCE, and higher GTP) on transcription. No attempt is made to check IB2 capping efficiency, as no capping will occur in the absence of the methyl donor, SAM.

Due to the costs of enzymes, it was not possible in this study to perform a comprehensive experiment that probes the significance of the RNA yields obtained from these highlighted reaction scenarios. However, to provide insight, we performed reactions in four replicates to compare the 2191 nt mRNA yield of formulated reaction buffers ± the *Vaccinia* capping enzyme system. A statistical comparison would enable us to determine if the presence of the capping enzyme in the integrated buffer significantly reduces mRNA yield. Our analysis shows no significant difference in yield, confirming that the capping enzyme in the formulated buffer does not substantially affect mRNA yield (Figure 2D, Tables S3–S5).

### Simple Platform for the Identification and Detection of Capped mRNA

In a strategy to develop an inexpensive integrated platform for the synthesis and analysis of mRNA, it was desirable to create a method that can rapidly identify mRNA and detect capped structures without the need for purification of the IVT product. Our strategy exploits RNase A’s specificity for single-stranded RNA (ssRNA) species under specific conditions. RNase A cleaves dsRNA species in low-saline solution^33^ and, by extension, the RNA strand on an RNA–DNA hybrid under this condition. However, as we have observed in experiments, it does not cleave dsRNA in a solution with excess salt. Moreover, DNA–RNA hybrids are more stable than DNA–DNA.^34^ Therefore, we reasoned that the DNA–RNA hybrid would remain intact and unaffected by the RNase A treatment under high saline conditions.

**Figure 3:**
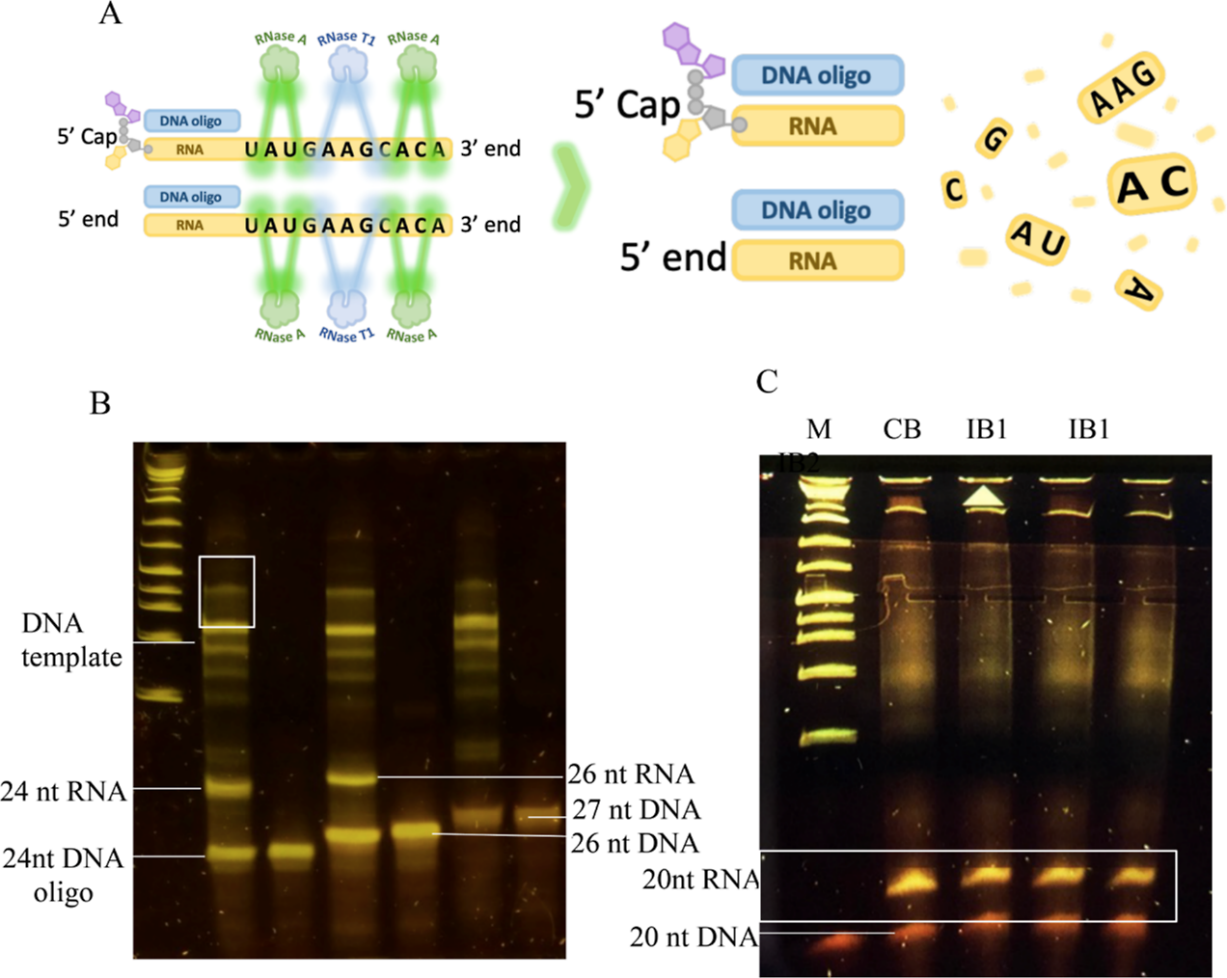
DNA primer-based protection assay and 8% urea-15% PAGE for mRNA analysis. (A) Workflow of the protection assay using a mixture of RNase A and T1. RNase A and T1 cleave after pyrimidine and guanidine residues of non-hybridized RNA (ssRNA), but the region hybridized to probe are left intact (B) lane 1: 86 nt RNA +5′ end complementary 24 nt DNA probe digested with RNase A; lane 2: 5′ end complementary 24 nt DNA oligo; lane 3: 86 nt RNA +5′ end complementary 26 nt DNA oligo digested with RNase A; lane 4: 26 nt 5′ end complementary DNA oligo; lane 5: 86 nt RNA + non-complementary 27 nt DNA oligo digested with RNase A; lane 6: non-complementary 27 nt DNA oligo. (C) IVT materials generated from CB, IB1 (in replicate), or IB2 + 20 nt DNA oligo digested with the RNase A and T1 mixture.

Consequently, oligonucleotides designed to anneal to any segment of a single-stranded RNA molecule will protect that region; conversely, mRNA sequence regions not hybridized to the oligonucleotide will be cleaved by RNase A. Therefore, when cleaved with RNase A, bands corresponding to the DNA probe and RNA fragment will be prominent on a denaturing PAGE. It is possible to resolve the RNA–DNA hybrid oligos on the PAGE because, first, under denaturing conditions, such as existing on the urea-PAGE, the oligo strands will separate. Second, we have observed in experiments that RNA and DNA oligos of the same length have different electrophoretic mobilities, with RNA migrating slower on a polyacrylamide gel. Therefore, DNA and RNA fragment of the same size can be separated. Lastly, we hypothesize if the RNA target is not present in a sample or the designed probe is not complementary to the target mRNA then only the DNA probe band will be observed. We propose that, on this basis, this assay can be used to confirm the identity of an mRNA. The general scheme of the Rnase A-based assay is illustrated in (Figure 3A).

To test our hypotheses, we designed a 24 and 26 nt DNA oligonucleotide (£2.5 per 50 nmoles) that anneals to the 5′ end of the target RNAs. This oligonucleotide was annealed to the RNA under saline conditions, and the reaction product was analyzed on 8% urea 8–20% polyacrylamide gel (urea-PAGE), as described in the Method. First, we tested the 24 nt DNA probe by hybridizing it to a target 86 nt RNA before RNase digest. Urea-PAGE analysis shows two prominent fragments corresponding to the oligonucleotide DNA probes and predicted 24 nt RNA fragments (Figure 3B, lane 1). The band corresponding to the DNA oligonucleotide is confirmed by comparison with the control 24 nt DNA probe sample run on a separate lane (Figure 3B, lane 2).

Similarly, the 26 nt DNA probe assay generated two prominent bands and a single band for treatment and control (26 nt DNA probe) samples, respectively (Figure 3B, lanes 3 and 4). The result shows that urea-PAGE resolves the 24 and 26 nt DNA probes from the 24 and 26 nt RNA oligos, respectively. We also note that the 24 and 26 nt RNA fragments migrate at markedly different rates, suggesting that urea-PAGE has a single-base resolution. Furthermore, we experimented with the same conditions using a 27 nt non-target DNA probe. As predicted, no band corresponding to the expected RNA fragment appears on the gel (Figure 3B, lane 5). The PAGE shows that if a probe is not complementary to RNA then RNA in the sample is completely digested. The result shows that no RNA fragment would be observed if synthesized RNA lacks perfect sequence complementarity with the designed DNA probe. Therefore, it demonstrates the assay’s utility in detecting synthesis and confirming the identity of target RNA in an IVT material. It could also be a quantitative tool by quantifying the amount of DNA probes hybridized to RNA.

The bands corresponding to the DNA fragments used for the IVT are highlighted in (Figure 3B). Note that the IVT product was not purified or DNase-treated before assay as it was unnecessary; therefore, DNA contaminants are expected. RNase A completely would digest all single-stranded RNA species in a sample. Therefore, other background nucleic acid bands are either DNA, DNA–RNA hybrids, or dsRNA species.

RNase T1 can be used for this assay; however, RNase A’s choice is primarily due to its cleaving specificity after cytidine-3′ and uridine 3′ monophosphates. Hence, the potential to generate smaller fragments, undetectable on PAGE, from the “unprotected” regions of the mRNA. In contrast, RNase T1 cleaves only after guanidine monophosphate and is likely to generate large fragments and may complicate analysis. It may involve identifying the RNase T1 sites on mRNA and predicting expected fragments by in silico analysis. However, any method that eliminates potential non-target RNA fragments requires no additional in silico analysis. Therefore, we reasoned that we could achieve complete digestion of contaminating non-target RNA fragments by utilizing an RNase T1 and RNase A mixture. Complete digestion of non-target mRNA regions is possible because C, U, and G nucleotides in these single-stranded RNA regions are cleaved by the enzyme mixture under saline conditions.

To test the effectiveness of this approach and its applicability to long mRNA, we digested 2191 nt mRNA synthesized under different reaction conditions (CB, IB1, and IB2) with the RNase A/T1 mixture under saline conditions (700 mM NaCl) and in the presence of a 20 nt probe. The urea-PAGE analysis of the digested samples reveals two prominent bands (see Figure 3C) corresponding to the predicted RNA fragment and the DNA probe. Other observed fainter bands and smears are DNA templates and possible DNA–RNA hybrids. Therefore, we show that the method can use an RNase A/T1 mixture in conjunction with designed probes to generate predicted RNA fragments.

The developed mRNA detection approach is flexible and enables the design of DNA oligonucleotides to target unique mRNA sequences and regions of any length. As a result, nuclease digest could generate unique RNA fragments and bands on PAGE. Moreover, targeting the 5′ end or the 3′ end of mRNA with an appropriate complementary DNA probe can detect capped mRNA and poly(A) tail, respectively.

### Single-Pot Capped mRNA Synthesis using the Integrated Buffer System

**Figure 4:**
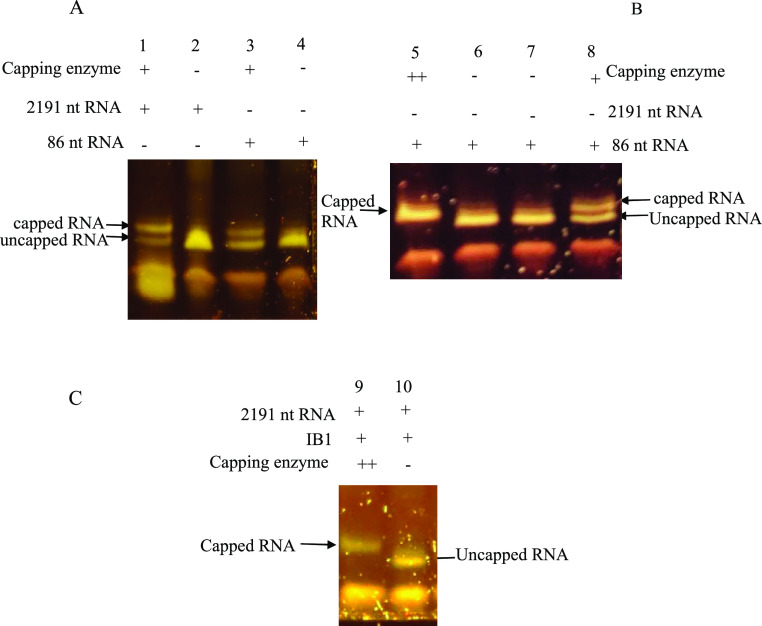
One-pot, two-step reactions in integrated buffer (IB1) and analysis by the DNA primer-based protection assay with urea-PAGE. Keys: + = present, – = absent, ++ = more capping enzyme added after 2 h incubation. All samples were analyzed by the digesting reactions product in the presence of a 20 nt oligo (protection assay) before 8% urea-15% PAGE) (A) lane 1 and 3: IVT-capping reaction was incubated at 37 °C for 2 h, followed by the protection assay and urea-PAGE. Lanes 2 and 4 (controls): assay set up under same conditions but catalyzed by only T7 RNA pol (B) assay set up with 86 nt RNA encoding DNA template. Lane 5: assay performed under the same conditions as (A, lane 3) but adding extra capping enzymes and incubating for another hour; Lane 6 (control): assay performed under the same conditions but without the capping enzymes. Lane 7 and 8: IVT-capping and control, respectively, incubated only for 2 h with no extra capping enzymes. (C) Lane 9 and 10: assay identical to lanes 5 and 6 but with a DNA template encoding 2191 nt mRNA used in the IVT-capping reaction.

To test the capping efficiency, we performed a single-step reaction in the developed integrated buffer containing T7 polymerase, *Vaccinia* capping enzyme, IVT, and capping components. First, we performed the *Vaccinia* enzyme system-based “co-transcription” capping reaction in the integrated buffer (containing buffer, IVT substrate, capping substrates, and capping enzymes) using a 2191 bp DNA template. The negative control has the same composition except for *Vaccinia* capping enzymes. Following the IVT-capping reaction, we treated the IVT product with a 20 nt DNA probe designed to protect the mRNA’s 5′ end, digested with RNase A, and analyzed by urea-PAGE analysis. The result shows two bands corresponding to the capped and uncapped mRNA (Figure 4A, lane 1) compared to the control that contains no capping enzymes (Figure 4A, lane 2). The result shows that both polymerase-catalyzed IVT and *Vaccinia* capping enzyme-mediated mRNA capping can take place simultaneously in this buffer. Here, T7 RNA polymerase and *Vaccinia* capping enzymes catalyze IVT and mRNA capping in a single system with no apparent inhibition of either transcription or capping activity. Capping mediated by the *Vaccinia* enzyme system requires a 5′ triphosphate end of an mRNA^35,36^ and inhibiting transcript synthesis, as observed in co-transcriptional incorporation of first-generation cap analogues, is not expected. Moreover, the full-length mRNA molecules, as observed in agarose gel analysis (Figure 2A,B), support the notion that actively transcribed mRNA is capped without disrupting transcription in this system. Notably, most capping enzymes act co-transcriptionally once the transcript reaches a length of 20–30 nucleotides.^18,37,38^

To establish if capping in this integrated system is affected by the RNA length, we performed a transcription-capping reaction using an 86 bp DNA. The sequences of the two DNA templates (2191 and 86 bp DNA) used in this experiment have identical 5′ end sequences and are targeted by the same 20 nt DNA probe. Similar to the assay performed for 2191 nt mRNA, the reaction product was digested with RNase A in the presence of the designed 20 nt DNA probe and analyzed on the urea-PAGE. The result shows two bands corresponding to capped and uncapped mRNA. The result for the 86 nt RNA is consistent with that obtained for the 2191 nt mRNA, demonstrating that the length of RNA does not significantly impact transcription and capping in this integrated system.

Although urea-PAGE analysis shows that a capping reaction occurred, it also revealed that the capping efficiency is not 100%. Visual inspection of the urea-PAGE estimated that about 70% 2191 nt and 50% 86 nt RNA were capped (Figure 4A). It was unclear why capping efficiency differed, as the RNA mass concentration of both samples was similar, and identical capping enzyme units were used in both reactions. However, a possible reason for the disparity could be the differences in the molar concentrations of these samples. Although the reactions generated similar mass concentrations for both transcripts, the shorter 86 nt sequence would have a higher molar concentration; therefore, the percentage of capped 86 nt would be negatively affected if capping rates were the same. What is clear is that the capping efficiency in both was not 100%. Therefore, optimizing the integrated buffer system was necessary to generate an entirely capped mRNA transcript.

### Optimization of the Integrated Buffer System to Enhance Capping Efficiency

To optimize capping efficiency in this system, we assumed that the *Vaccinia* capping enzyme loses efficiency with longer incubation time. A previous study has shown that longer incubation increases capping, but the enzyme loses activity for hours,^21^ which is consistent with our assumption. The IVT/capping reaction was performed over 3 h, and we reasoned that much of the earlier transcripts would have been effectively capped (within 1–2 h) than those synthesized much later in the reaction. In addition, we also speculated that the amount of capping enzyme used might not be sufficient. It is also possible that buffer conditions or the release of by-products, such as pyrophosphates, during the reaction may have impacted the capping enzyme activity.

We first tested the efficiency of capping the 86 nt transcript in this experiment with a control experiment (reaction without capping enzyme). We performed the reaction as before to test our hypothesis that insufficient enzyme concentration and loss of enzyme activity may be responsible. However, we supplemented the reaction with more capping enzymes in the last hour. A comparison of these two, following urea-PAGE analysis, reveals a mobility shift, with a fragment from the capping reaction (Figure 4B, lane 5) migrating slower on the gel than on the negative control (Figure 4B, lane 6). Another control was performed under identical conditions as the first reaction but with no enzyme supplementation in the last hour (Figure 4B, lane 8) with an appropriate negative control (Figure 4B, lane 7). In contrast with the initial reaction (Figure 4A), more efficient capping was observed when the enzyme was increased. The result shows that supplementing the reaction with more enzymes in the last hour enabled 100% capping efficiency. Similarly, the same experiment was performed with the 2191 nt enzyme. The result (Figure 4C) demonstrates 100% capping of the mRNA product.

The result demonstrates the successful integration of IVT and capping processes mediated by the *Vaccinia* capping enzyme into the one-buffer system.

### Further Tests to Demonstrate the Versatility of Capping and Assay Methods

**Figure 5:**
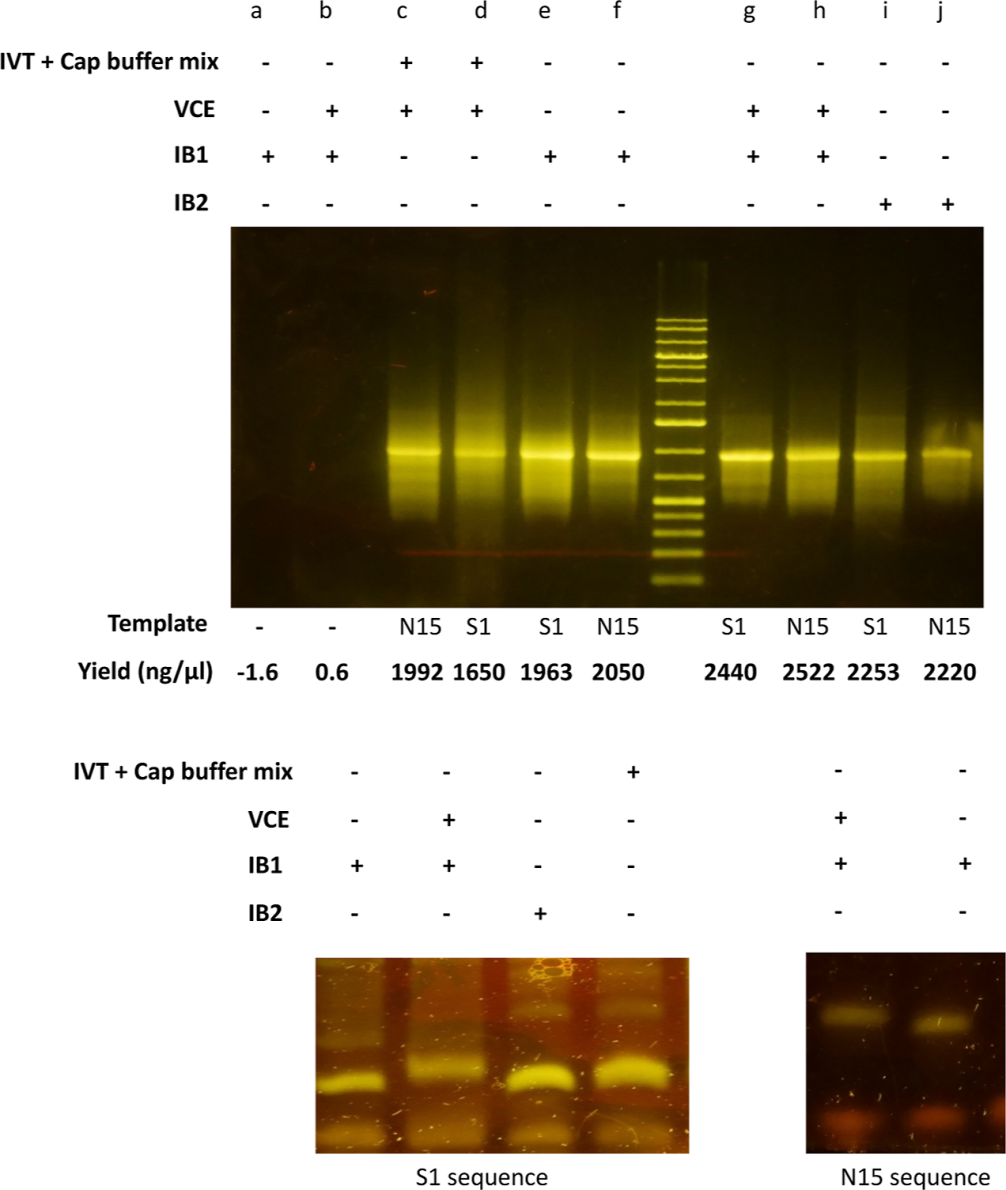
Further evaluation of the applicability of methods using additional DNA sequence constructs (N15 and S1 DNA sequences). (A) Agarose gel electrophoresis of RNA samples generated under conditions specified on the gel labels. RNA transcription and capping under different, (B) RNA protection assay using a 17 nt probe and urea-PAGE analysis of S1 RNA generated from the different reaction buffers, and (C) assay using a 20 nt DNA probe and urea-PAGE analysis of capped and uncapped N15 RNA generated under conditions indicated on the gel.

To further demonstrate the versatility of developed methods, we performed the experiments on two additional DNA constructs denoted as N15 (1024 bp) and S1 (1152 bp) sequences (see Figure S2). First, we transcribed them in IB1, IB2, and commercial VCE + IVT reaction buffer mix (1:1 mix) before agarose gel electrophoresis. To test if the developed buffer generates spurious products, no template control was performed in the IB1 buffer with or without VCE. The result shows that transcription occurred across all tested conditions except for the no-control template (see Figure 5A). Interestingly, the 1 VCE:1 IVT mix used as control also generated transcripts. As expected, no RNA product was generated in the no template control. Again, consistent with earlier experiments, the result suggests no significant differences in the yield between the designed buffer and the commercial 1 VCE:1 IVT mix. The results demonstrate that the developed reaction buffer supports transcription irrespective of the sequence.

Following transcription, the S1 and N15 RNA samples generated under different conditions were subjected to the DNA-probe RNase protection assay before UREA PAGE analysis. From experience in our lab, we have observed optimal single-nucleotide resolution in the range of 16–24 nt DNA oligos on our urea-PAGE. We reasoned that RNA fragments within this range would be optimally resolved in our system. Therefore, our choice of oligonucleotides ranging from 16 to 20 nt in length was to ensure optimal single-nucleotide resolution while demonstrating probe design flexibility for cap analysis in our system. We anticipate that single-nucleotide resolution of shorter or longer oligonucleotide fragments is also possible with optimal electrophoresis run time and urea-PAGE composition. Consistent with previous results, expected RNA fragments corresponding to capped and uncapped sequences were observed (see Figure 5B,C). Taken together, the result demonstrates the applicability of the capping and assay methods on different sequences.

This study presents a less expensive single-step method that combines the advantages of both co-transcriptional and enzymatic capped mRNA synthesis. We developed this novel one-step platform for capped mRNA synthesis by rationally designing and optimizing an “integrated reaction buffer” that supports both IVT and *Vaccinia* enzyme-mediated capping. This approach eliminates the extra unit operation and attendant costs of performing two-step synthesis and purification steps in the traditional enzymatic method while retaining high capping efficiency. We have decreased the complexity by reducing the synthetic steps, thereby enhancing scalability and manufacturability. We show that the system produces mRNA yields similar to those of commercial IVT kits.

Additionally, we report a simple, novel method for rapidly and quantitatively detecting mRNA and the capped structure. This approach involves protecting a short target sequence within mRNA with a complementary DNA probe, followed by RNase digest and one-nucleotide resolving urea-PAGE. This platform integrates a novel one-step synthesis of capped mRNA with a one-step assay that, in conjunction with PAGE, identifies mRNA and structural RNA modifications. The assay uses inexpensive reagents: DNA primers (£2 per 100 μM per 300 assays) and susceptible dyes (thiazole orange; £1 per 5 mg per 250 assays). It does not require costly labeled RNA probes or any tedious purification steps. However, adequate precaution is necessary for handling RNase during the protection assay to avoid accidental contamination of samples.

We speculate that this method could be deployed for mass spectrometry analysis of mRNA by designing different oligos targeting different regions spanning the length of the mRNA. It may also find applications in isolating and detecting post-transcriptional modifications in biological samples by designing oligonucleotide primers targeting the modified sequence in the mRNA in conjunction with mass spectrometry analysis. We predict that, if implemented, this would achieve higher sequence coverage than is currently possible using traditional RNase-mass mapping approaches.

The limitation of this study is that only cap 0 capping efficiency was tested in the developed reaction buffer. Cap 0 is currently being phased out due to potential immunogenicity concerns. We speculate that the developed buffer will also support Cap 1 capping since the same reaction buffer supports commercial VCE and mRNA Cap 2′-*O*-methyltransferase activities. In the future, we hope to test this and further optimize reaction conditions to improve RNA and capping yield.

## Methods

Q5 High-Fidelity DNA polymerase, dNTPs, and designed primers from MWG Eurofins were used for PCR and IVT performed using T7 RNA polymerase (New England Biolabs), *Vaccinia* Capping System (New England Biolab), and synthetic gene from GeneArt Gene Synthesis (Invitrogen Life technologies). Two synthetic genes encoding 86 nt RNA and 2191 nt mRNA were used for IVT (Figure S1). The DNA template sequence architecture, primer sequences, and probes used in the DNA primer-based protection assay are depicted in Figure S1. Four DNA primers purchased from Thermo Fisher Scientific were used in the protection assay. The NTP set, 100 mM solution (ThermoFisher Scientific), and the HiScribe T7 High Yield RNA Synthesis Kit were used for IVT. 50×TAE and 10×TBE buffers from Thermo Fisher Scientific were used to perform agarose and urea-PAGE, respectively. Other chemicals used include urea pellets (Sigma-Aldrich), molecular grade agarose (Bioline), acrylamide/bis-acrylamide (Sigma-Aldrich), and nuclease-free water (Thermo Fisher Scientific). Thiazole orange (Sigma-Aldrich) was used as a dye for urea-PAGE. Midori green direct (Nippon genetics) was used as a stain for the RNA agarose gel electrophoresis and analysis. A Fastgene 470 nm blue LED transilluminator (Nippon Genetics) was used to image all gels.

### Design and Preparation of an Integrated Buffer

IVT buffers were extracted from eleven literature refs (39)–^40^^41^^42^^43^^44^ and manufacturers’ manuals (Affymetrix, Sigma-Aldrich, Promega, NEB, and Thermo Fisher). Their chemical component concentrations were compared and analyzed to obtain median concentrations. Similarly, a search identified the components of *Vaccinia* capping buffer from the literature^21,45,46^ and manufacturing sources (Hongene, Biotech, and NEB) and analyzed them. The final components and concentration of the integrated buffers were selected by applying three rationalized rules to the data: (1) include a chemical if it is essential for one reaction type even if it is not present in the other. It is essential when a chemical occurs at 100% frequency in the buffer composition found in the literature. (2) exclude if a chemical is not essential and not found in the other reaction. (3) Rule of integration: use the highest concentration value for chemicals that occur in both unless it is detrimental to the other buffer or evidence exists that the lowest quantity has the same effect as the highest value, in which case use the lowest value*. Essential was defined as when a chemical occurs at 100% frequency in a buffer. By applying these rules, the chemical components and the concentration of the predicted integrated buffer were established. 10× Integrated buffers were prepared by adding each component at the right concentration deduced from the analysis of the concentration data obtained from the literature. The buffer composition can be broadly classified as IVT-CAP core components (consisting of TRIS, MgCl_2_, DTT, spermidine, and NTPs) and capping substrates (SAM and GTP). Two versions of the 10× integrated buffer (IB) were prepared: (1) Integrated buffer 1 (IB1) is composed of 500 mM Tris, 99 mM MgCl_2_, 10 mM DTT, 18 mM spermidine, 20 mM NTPs, 1 mM SAM, and 5 mM GTP (25 mM GTP in total). (2) Integrated buffer 2 (IB2) of 500 mM Tris, 99 mM MgCl_2_, 10 mM DTT, 18 mM spermidine, and 20 mM NTPs. 1× solution, when required, is obtained by dilution with nuclease-free water. Stock solutions of all the required components for an integrated buffer were prepared in sterile 15 mL polystyrene falcon tubes (brand name) using nuclease-free water (Thermo Fisher Scientific). These stock solutions were filtered with single-use 0.2 μm PES syringe filters to minimize adventitious contamination and stored at the appropriate temperature recommended by manufacturers.

### In Vitro Transcription Procedure

An 86 and 2161 bp DNA encoding mRNA was amplified from the 86th and 2161st regions of a plasmid (termed pSpmep2) and used as the template for the in vitro reaction. PCR was performed using primers flanking the dsRNA gene under the following conditions. 0.02 U/μL Q5 High-Fidelity DNA Polymerase, 200 μM dNTPs, 0.5 μM each of forward and reverse primers, and 10 ng of DNA template. The following PCR parameters were used: the initial denaturation was 1 cycle of 30 s at 97 °C, followed by 30 cycles of (30 s at 97 °C, 30 s at 58 °C, and 30 s at 72 °C) and a final extension at 72 °C for 2 min. IVT was performed using T7 RNA polymerase (New England Biolabs). For IVT using CB (HiScribe T7 High Yield RNA Synthesis Kit, NEB), a 20 μL of reaction was set up and contained 10 mM NTPs, 1× reaction buffer, 1 μg of DNA template, and 2 μL of T7 RNA polymerase in 20 μL of RNase-free water. IVT reactions using a formulated “integrated” buffer were prepared as follows: 20 μL of reaction was set up by mixing 1 μg of the DNA template (dissolved in 10 μL of nuclease-free water) with 2 μL of 10× integrated buffer. Then, an appropriate volume of nuclease-free water and 2 μL of T7 polymerase (NEB) was added to make the final 1× reaction solution.

### mRNA Capping Method

Enzymes from the kit, *Vaccinia* Capping System (NEB), were used to perform a capping reaction in the “integrated” buffer and benchmarked against the reaction in the *Vaccinia* capping buffer (NEB). Four distinct reactions were performed: (1) 20 μL capping reaction using a commercial kit: heat 10 μg of RNA (purified 86 or 2191 nt) in 15.0 μL of nuclease-free water at 65 °C for 5 min. Place the tube on ice for 5 min and add the following: 2 μL of 10× capping buffer, 1 μL of GTP (10 mM), 1 μL of SAM (2 mM, dilute 32 mM stock to 2 mM), and 1.0 μL of *Vaccinia* capping enzyme (NEB). (2) Standalone capping reactions in “integrated” buffers 1 and 2: 5 μL of IVT product (containing an estimated 70–90 μg of RNA) in 1× integrated buffer was combined with 13 μL integrated buffer and 2 μL of *Vaccinia* capping enzymes (NEB). (3) Integrated (single-step) IVT and capping reaction: 1 μg of the DNA template (dissolved in 15 μL nuclease-free water was mixed with 2 μL of T7 polymerase (NEB) and 2 μL of *Vaccinia* capping enzymes (NEB). (4) Integrated (single-step) IVT and capping reaction with supplementary capping enzyme: 1 μg of the DNA template (dissolved in 15 μL of nuclease-free water) was mixed with 2 μL of T7 polymerase (NEB) and 2 μL of *Vaccinia* capping enzymes (NEB). All reactions were incubated at 37 °C for 2 or 3 h; reaction time depends on the experimental design. For reactions incubated for 3 h, the reaction was supplemented with 2 μL of *Vaccinia* capping enzyme (NEB) added after 2 h (additional treatment) or not (control for treatment) before incubation for another 1 h.

### RNA Purification

IVT-generated RNA was made with 50 μL of nuclease-free water, treated with 2 μL of DNase I, and incubated at 37 °C for 20 min 80 μL of 5 M NaCl and 150 μL of isopropanol were added to this mixture before transfer to a silica spin column (Geneflow Limited). The spin column was centrifuged at 13,000 rpm for 30 s, and the flowthrough was discarded. 500 μL 70% ethanol was added, and the spin column was centrifuged at 13,000 rpm for 30 s. The supernatant was discarded, followed by repeated centrifugation for 30 s to remove residual ethanol. The column was eluted with 100 μL of nuclease-free water. The RNA concentration and quality were determined using a NanoDrop 2000c spectrophotometer (Thermo Scientific).

### DNA Probe-based RNase Protection Assay for mRNA Detection and 5′ Cap Analysis

1 μg (for 86 nt RNA) or 5 μg (>1000 nt RNA) of purified and un-purified IVT-generated mRNA was mixed with 800 ng of either 20, 24, or 26 nucleotide DNA primer targeting the 5′ end of RNA. For cap analysis, DNA oligos of 16–20 nt length are designed to enable single-nucleotide resolution without prolonged run time. Following annealing, 10 μL of 700 mM NaCl solution was added. A non-target 27 nucleotide DNA was used in the control assay. 2 μg of RNase A or a mixture of RNase A (2 μg) and T1 (2 U) (Thermo Fisher Scientific) was added and incubated for 15 min. After the RNase digest, the sample was loaded on 8–20% urea-PAGE (10.1 × 8.2 cm × 1 mm gel) and run for 2 h at 200 V. The gel was stained with thiazole orange (10 ng/mL) solution in 1× TBE buffer. The gel was imaged using a 470 nm blue LED transilluminator (Fastgene).

### Agarose Gel Electrophoresis

1% agarose gel was used for gel electrophoresis. RNA loading dye 2× (NEB) was added to the RNA sample and loaded on the gel. 1× TAE buffer (40 mM Tris (pH 7.6), 20 mM acetic acid, and 1 mM EDTA) was used to perform electrophoresis at 100 V for 45 min. The gel was imaged using a 470 nm blue LED transilluminator (Fastgene).

### Statistical Analysis

The experiment subjected to statistical analysis was conducted in quadruplicate. Statistical analysis was accomplished using Graphpad Prism 9. One-way analysis of variance (ANOVA) and Tukey’s multiple comparison analysis were performed to determine if there were significant differences between the different conditions tested.

## Supporting Information Available

Architecture of the DNA template amplified by PCR and transcribed to generate 86 and 2161 nt mRNA; literature review of the chemical components of both *Vaccinia* capping and IVT reaction buffers and the number of references that mention specific chemicals; chemical concentrations of integration buffer (IB1) obtained by rational design; RNA yield of reactions set up in integrated buffer (IB1) with or without the *Vaccinia* Capping System and performed in four replicates; descriptive statistics (one-way ANOVA) for RNA yield from the transcription of DNA templates in IB1 buffer under defined conditions (IVT reaction in IB1 ± capping enzyme for both RNA-encoding DNA templates); and two-way ANOVA summary table for the effect of induction time on AREV4 dsRNA yield from different formulated media (PDF)
