# Supplementary material for: Low Resource Integrated Platform for Production and Analysis of Capped mRNA
Source: ACS Synth Biol. 2022 Dec 10;12(1):329–39. doi: 10.1021/acssynbio.2c00609 (PMC9872168; doi:10.1021/acssynbio.2c00609)
Supplement: Supplementary file 1 — sb2c00609_si_001.pdf [file sb2c00609_si_001.pdf]

Supporting Information

Low resource, integrated platform for production and analysis of capped mRNA

Alison Obinna Nwokeoji[1*], Tachung Chou[2,3], Eleojo Ahuva Nwokeoji[3]

***Correspondence: a.nwokeoji@sheffield.ac.uk**

1 Chemical and Biological Engineering, S1 3JD University of Sheffield, Sheffield, South Yorkshire, United Kingdom.

2 School of Biosciences, S10 2TN, University of Sheffield, Sheffield, South Yorkshire, United Kingdom.

3 All First Technologies, No. 208, Longnan Rd, Pingzhen Dist, Taoyuan City 324, Taiwan

SUPPLEMENTARY INFORMATION

Supplementary Figures:

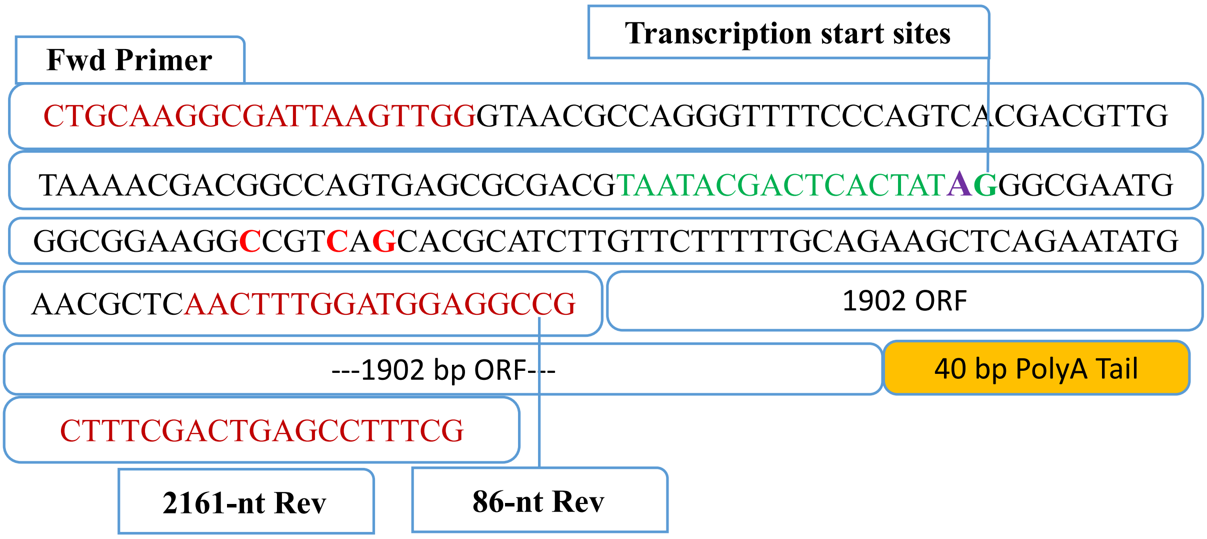

Figure S1 Architecture of DNA template amplified by PCR and transcribed to generate 86 nt and 2161 nt mRNA. The primers are indicated. 86-nt Rev: reverse primer sequence for the template encoding 86-nt RNA. 2161-nt Rev: reverse primer sequence for the template encoding 86-nt RNA. Both template constructs were amplified using one forward primer (labelled Fwd primer). Sequence highlighted in green: T7 RNA promoter sequence. The DNA probe sequences used in the RNase protection assay are 20, 22 and 26 nt in length, starting from the bold purple letter (A) at the 5' end to the bold red letters at the 3' end. B)

Supplementary Tables:

Table S1. *The literature review of the chemical components of both Vaccinia capping and IVT reaction buffers and the number of references that mention specific chemicals.*

Component	No. of references that mention component/ Total no. of references
**Tris-HCl**	Capping buffer: 5/5, Transcription buffer: 11/11
**$MgCl_2$**	Capping buffer: 5/5, Transcription buffer: 11/11

**DTT**	Capping buffer: 5/5, Transcription buffer: 11/11
**KCl**	Capping buffer: 5/5, Transcription buffer: 1/11
**NaCl**	Transcription buffer: 6/11
**Spermidine**	Transcription buffer: 11/11
**Triton X-100™**	Transcription buffer: 3/11

Table S2. The chemical concentrations of Integration buffer (IB1) obtained by rational design

**Integrated Buffer component**	**Tris**	**$MgCl_2$**	**DTT**	**Spermidine**	**NTPS**	**SAM**	**GTP**
Conc. (mM)	50	9.9	1*	1.8	2.0	0.1	0.5

Table S3 *RNA yield of reactions set up in integrated buffer (IB1) with or without Vaccinia capping system and performed in four replicates*

Biological replicates	2191 nt Spmep		86 nt Spmep	
	No capping system Yield (ng/µl)	capping system Yield (ng/µl)	No capping system Yield (ng/µl)	capping system Yield (ng/µl)
1	2030	1540	1880	1920
2	1371	2020.3	1672	2050

3	1700	1756	1920	1950
4	1400	1709	1760	2112

Table S4 *Descriptive statistics (one-way ANOVA) for RNA yield from the transcription of DNA templates in IB1 buffer under defined conditions (IVT reaction in IB1 + or - capping enzyme for both RNA-encoding DNA templates)*

**Tukey's multiple comparisons test**	**Mean Diff.**	**95.00% CI of diff.**	**Signifi-cant?**	**Summary**	**Adjusted P Value**	
2191 nt (no cap) vs 2191 nt (cap)	-131.1	-544.7 to 282.5	No	ns	0.7840	A-B
2191 nt (no cap) vs 86 nt (no cap)	-182.8	-596.3 to 230.8	No	ns	0.5732	A-C
2191 nt (no cap) vs 86 nt (cap)	-382.8	-796.3 to 30.83	No	ns	0.0730	A-D
2191 nt (cap) vs 86 nt (no cap)	-51.68	-465.3 to 361.9	No	ns	0.9817	B-C
2191 nt (cap) vs 86 nt (cap)	-251.7	-665.3 to 161.9	No	ns	0.3169	B-D

86 nt (no cap) vs 86 nt (cap)	-200.0	-613.6 to 213.6	No	ns	0.5025	C-D		
Test details	Mean 1	Mean 2	Mean Diff.	SE of diff.	n1	n2	q	DF
2191 nt (no cap) vs 2191 nt (cap)	1625	1756	-131.1	139.3	4	4	1.331	12
2191 nt (no cap) vs 86 nt (no cap)	1625	1808	-182.8	139.3	4	4	1.855	12
2191 nt (no cap) vs 86 nt (cap)	1625	2008	-382.8	139.3	4	4	3.886	12
2191 nt (cap) vs 86 nt (no cap)	1756	1808	-51.68	139.3	4	4	0.5246	12
2191 nt (cap) vs 86 nt (cap)	1756	2008	-251.7	139.3	4	4	2.555	12
86 nt (no cap) vs 86 nt (cap)	1808	2008	-200.0	139.3	4	4	2.030	12

Table S5 *Two-way ANOVA Summary Table for the effect of induction time on AREV4 dsRNA yield from different formulated media*

ANOVA table	SS	DF	MS	F (DFn, DFd)	P value
Treatment (between columns)	303086	3	101029	F (3, 12) = 2.603	P=0.1002

Residual (within columns)	465726	12	38810
Total	768812	15	

N15 DNA sequence

Transcription start sites

Fwd Primer

TATAGGGCGAATTGGCGGAACCATGGGTTAATACGACTCACTATAGGG

AGAAAATGTCTGATAATGGACCCCAAAATCAGCGAAATGCACCCCGCATTACGTTTG
GTGGACCCTCAGATTCAACTGGCAGTAACCAGAATGGAGAACGCAGTGGGGCGCGA
TCAAAACAACGTCGGCCCCAAGGTTTACCCAATAATACTGCGTCTTGGTTCACCGCTC
TCACTCAACATGGCAAGGAAGACCTTAAATTCCCTCGAGGACAAGGCGTTCCAATTA
ACACCAATAGCAGTCCAGATGACCAAATTGGCTACTACCGAAGAGCTACCAGACGAA
TTCGTGGTGGTGACGGTAAAATGAAAGATCTCAGTCCAAGATGGTATTTCTACTACCT
AGGAACTGGGCCAGAAGCTGGACTTCCCTATGGTGCTAACAAAGACGGCATCATATG
GGTTGCAACTGAGGGAGCCTTGAATACACCAAAAGATCACATTGGCACCCGCAATCC
TGCTAACAATGCTGCAATCGTGCTACAACTTCCTCAAGGAACAACATTGCCAAAAGG
CTTCTACGCAGAAGGGAGCAGAGGCGGCAGTCAAGCCTCTTCTCGTTCCTCATCACG
TAGTCGCAACAGTTCAAGAAATTCAACTCCAGGCAGCAGTAGGGGAACTTCTCCTGC
TAGAATGGCTGGCAATGGCGGTGATATAGGGCGAATTGGCGGAATGCTGCTCTTGCTT
TGCTGCTGCTTGACAGATTGAACCAGCTTGAGAGCAAAATGTCTGGTAAAGGCCAAC
AACAACAAGGCCAAACTGTCACTAAGAAATCTGCTGCTGAGGCTTCTAAGAAGCCTC
GGCAAAAACGTACTGCCACTAAAGCATACAATGTAACACAAGCTTTCGGCAGACGTG
GTCCAGAACAAACCCAAGGAAATTTTGGGGACCAGGAACTAATCAGACAAGGAACT
GATTACAAACATTGGCCGCAAATTGCACAATTTGCCCCCAGCGCTTCAGCGTTCTTCG
GAATGTCGCGCATTGGCATGGAAGTCACACCTTCGGGAACGTGGTTGACCTACACAG
GTGCCATCAAATTGGATGACAAAGATCCAAATTTCAAAGATCAAGTCGGATCCACTCC
CTTCCTGATGGACCTGGA

Rev primer

S1 DNA sequence

Transcription start sites

Fwd Primer

TATAGGGCGAATTGGCGGAACCATGGGTTAATACGACTCACTATAGGG

AGACACGCCTAAACGAACATGAAATTTCTTGTTTTCTTAGGAATCATCACAACTGTAG
CTGCATTTCACCAAGAATGTAGTTTACAGTCATGTACTCAACATCAACCATATGTAGTT
GATGACCCGTGTCCTATTCACTTCTATTCTAAATGGTATATTAGAGTAGGAGCTAGAAA
ATCAGCACCTTTAATTGAATTGTGCGTGGATGAGGCTGGTTCTAAATCACCCATTCAG
TACATTGATATCGGTAATTATACAGTTTCCTGTTTACCTTTTACAATTAATTGCCAGGAA
CCTAAATTGGGTAGTCTTGTAGTGCGTTGTTCGTTCTATGAAGACTTTTTAGAGTATCA
TGACGTTCGTGTTGTTTTAGATTTCATCTAAACGAACAAACTAAAATGTCTGATAATG
GACCCCAAAATCAGCGAAATGCACCCCGCATTACGTTTGGTGGACCCTCAGATTCAA
CTGGCAGTAACCAGAATGGAGAACGCAGTGGGGCGCGATCAAAACAACGTCGGCCC
CAAGGTTTACCCAATAATACTGCGTCTTGGTTCACCGCTCTCACTCAACATGGCAAGG
AAGACCTTAAATTCCCTCGAGGACAAGGCGTTCCAATTAACACCAATAGCAGTCCAG
ATGACCAAATTGGCTACTACCGAAGAGCTACCAGACGAATTCGTGGTGGTGACGGTA
AAATGAAAGATCTCAGTCCAAGATGGTATTTCTACTACCTAGGAACTGGGCCAGAAG
CTGGACTTCCCTATGGTGCTAACAAAGACGGCATCATATGGGTTGCAACTGAGGGAG
CCTTGAATACACCAAAAGATCACATTGGCACCCGCAATCCTGCTAACAATGCTGCAAT
CGTGCTACAACTTCCTCAAGGAACAACATTGCCAAAAGGCTTCTACGCAGAAGGGAG
CAGAGGCGGCAGTCAAGCCTCTTCTCGTTCCTCATCACGTAGTCGCAACAGTTCAAG
AAATTCAACTCCAGGCAGCAGTAGGGGAACTTTTCCTGCTAGAATGGCTGGCAATGG
CGGGATCCACT CCCTTCCTGATGGACCTGGA

Rev primer

Figure S2 Architecture of N15 and S1 DNA sequences amplified by PCR and transcribed to generate 1104 nt and 979 nt RNA. The primers are indicated. Sequence highlighted in green: T7 RNA promoter start site. The DNA probe sequences used in the RNase protection assay for N15 and S1 are 20 nt and 17 nt in length, respectively, starting from the bold purple letter (A) at the 5' end to the bold red letters at the 3' end.
